# Structural and molecular correlates of cognitive aging in the rat

**DOI:** 10.1038/s41598-019-39645-w

**Published:** 2019-02-14

**Authors:** Cristina Mota, Ricardo Taipa, Sofia Pereira das Neves, Sara Monteiro-Martins, Susana Monteiro, Joana Almeida Palha, Nuno Sousa, João Carlos Sousa, João José Cerqueira

**Affiliations:** 1 Life and Health Sciences Research Institute (ICVS), School of Medicine, University of Minho, Braga, Portugal; 2 ICVS/3B’s - PT Government Associate Laboratory, Braga/Guimarães, Portugal

## Abstract

Aging is associated with cognitive decline. Herein, we studied a large cohort of old age and young adult male rats and confirmed that, as a group, old  rats display poorer spatial learning and behavioral flexibility than younger adults. Surprisingly, when animals were clustered as good and bad performers, our data revealed that while in younger animals better cognitive performance was associated with longer dendritic trees and increased levels of synaptic markers in the hippocampus and prefrontal cortex, the opposite was found in the older group, in which better performance was associated with shorter dendrites and lower levels of synaptic markers. Additionally, in old, but not young individuals, worse performance correlated with increased levels of BDNF and the autophagy substrate p62, but decreased levels of the autophagy complex protein LC3. In summary, while for younger individuals “bigger is better”, “smaller is better” is a more appropriate aphorism for older subjects.

## Introduction

Aging is a process that, even in healthy individuals, is generally linked to a decline in cognitive abilities^1^. However, one of the most striking characteristics of human aging is its heterogeneity^2,3^, with some individuals maintaining a preserved cognitive function until late in life. Since cognitive ability is a crucial determinant of elderly people’s quality of life, a thorough understanding of the mechanisms underlying its heterogeneity is of paramount importance. To this purpose, we decided to study a large cohort of young adults (4–6 month-old) and older rats (22–24 month-old).

Rats have been intensively used as models of cognitive aging^4–7^. A large body of literature indicates that, as in humans, spatial learning and memory tasks in rodents also require the hippocampus (HPC) and the medial prefrontal cortex (mPFC), and typically display performance decrements across the lifespan^5,6,8^. In fact, older male rats, approximately 22–24 months-old, show impairments in several spatial memory tasks including: Y and T mazes^9^, radial arm maze^10^, Morris water maze^6–8^, water radial arm maze^11^ and Barnes maze^12^.

The well-documented age-related behavioral deficits are concomitant, and seem to be correlated with morphological alterations in brain structure. It is widely accepted that aging is accompanied by an overall brain volume loss, in both humans^13–16^ and rats^17,18^, that accompanies the decline in cognitive function. Moreover, several studies reported age-related cognitive decline to be associated with volume loss and dendritic atrophy in areas implicated in cognitive abilities, such as the HPC and the mPFC^13,18–21^.

The homeostasis of the mammalian neuroarchitecture is a dynamic process involving a balance between sprouting and pruning. The mechanisms underlying these processes are particularly active during development and pathological neurodegeneration^22,23^ but are also functional in physiological conditions. Of note, while in equilibrium, the relative importance of each of these processes varies throughout the lifespan, with synapse and dendritic formation generally exceeding pruning during brain development, and an opposite trend occurring in the adult brain^23,24^. Importantly, the maintenance of this balance is under tight control through protein synthesis and autophagic recycling^23–26^.

Herein, we explored cognitive aging and its structural and molecular correlates in a large set of old and young male Wistar Han rats. The results show that while for younger individuals “bigger is better”, it seems that “smaller is better” is more appropriate for older subjects, as older animals with smaller dendritic trees, increased neuronal autophagy and decreased brain-derived neurotrophic factor (BDNF) and synaptic markers, presented the best performances.

## Results

### Age is associated with cognitive decline and behavioral heterogeneity

**Figure 1:**
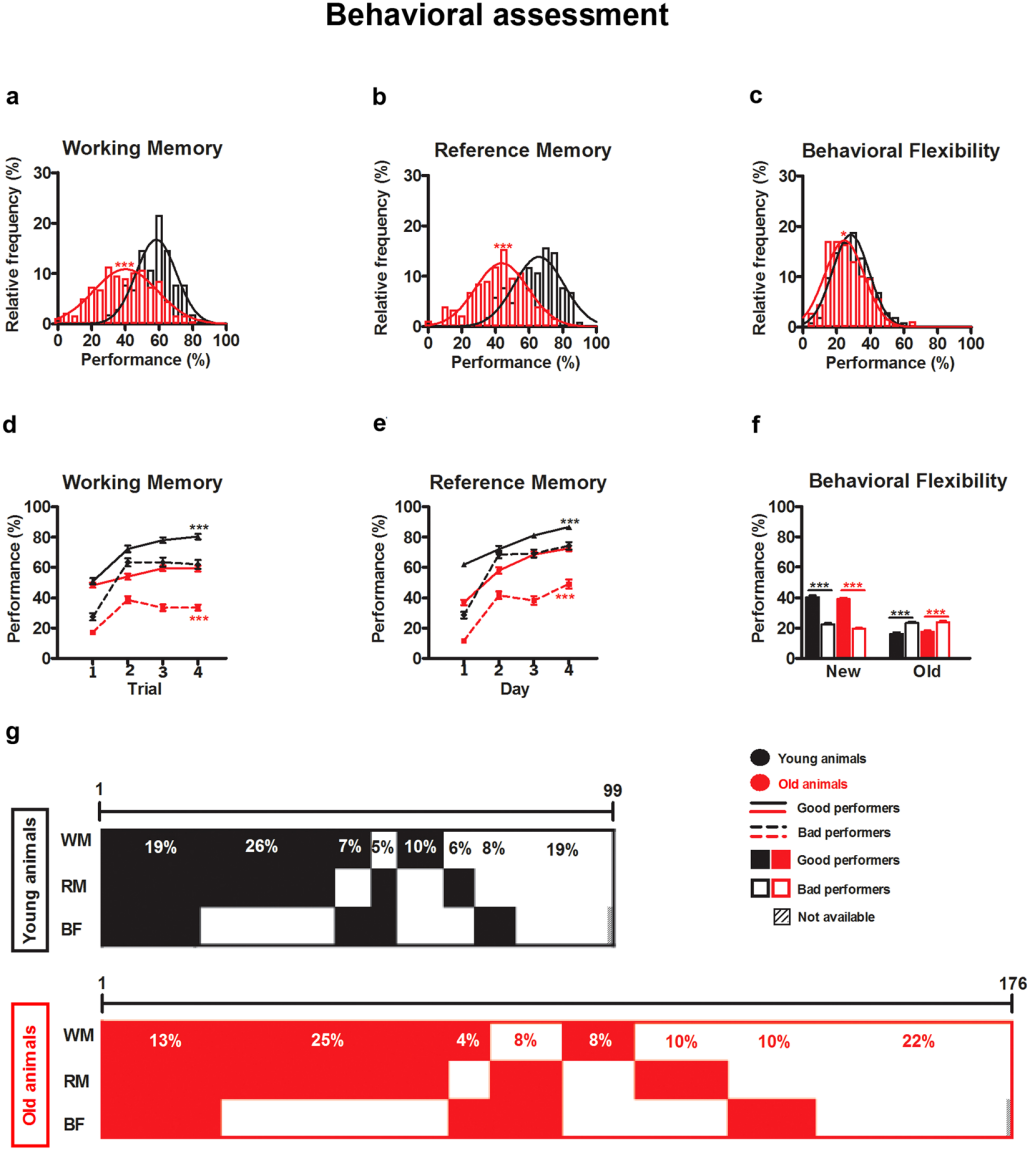
Behavioral assessment and performance clustering of younger and older rats. When all animals are considered: (**a**) The working memory (older n = 176; younger n = 102) and (**b**) reference memory (older n = 176; younger n = 102) PIs of older rats are worser and broader than that of youngsters. (**c**) The performance in behavioral flexibility (older n = 176; younger n = 101) is less variable but maintains the previous trend as older rats are the worst performing group. When similar age animals are clustered (see methods for details) in GPs and BPs: GP and BP clusters had significantly different learning curves in (**d**) working memory (older: GPs n = 89 BPs n = 87; younger: GPs n = 63 BPs n = 39) and (**e**) reference memory tasks (older: GPs n = 99 BPs n = 77; younger: GPs n = 61 BPs n = 41). (**f**) GPs spent more time in the new and less in the old quadrants of the behavioral flexibility task (older: GPs n = 62 BPs n = 114; younger: GPs n = 40 BPs n = 61). Interestingly, (**g**) the frequencies of the different patterns were similar in younger and older animals. Continuous lines in (**a**–**c**) are Gaussian fits. Error bars represent SEM; *p < 0.05; ***p < 0.001.

Older animals displayed a worse cognitive performance in all tested domains (working memory: *t*(267) = −10.122, *p* < 0.0005, *d* = 1.148; reference memory: *t*(276) = −11.274, *p* < 0.0005, *d* = 1.403; behavioral flexibility: *t*(275) = −2.222, *p* = 0.027, *d* = 0.277) (Fig. 1a–c). Moreover, the performance of older rats in working and reference memory was more heterogeneous than that of younger rats (working memory variance: older = 290.33%^2^ vs. younger = 141.12%^2^; reference memory variance: older = 262.47%^2^ vs. younger = 180.47%^2^), while no differences were observed in the distributions of behavioral flexibility performances of older and younger animals (variance: older = 128.96%^2^ vs. younger = 118.56%^2^) (Fig. 1a–c).

A k-means clustering was performed to classify younger and older animals according to their performance in working memory, reference memory or behavioral flexibility tasks. This resulted, for each test and age, in two groups of subjects (good performers – GPs and bad performers – BPs), which had significantly different performances (Table 1, Fig. 1d–f; see also the Supplementary Fig. S1 for the escape latency to find the platform (s)). A two-way ANOVA revealed a significant effect of age and performance, as a significant interaction between these two factors, both in the working and reference memory tests (Table 1, Fig. 1d–f). Overall, in memory tests, younger adult rats performed better than older rats, with younger GPs being the best (Tukey’s test p < 0.001), younger BPs and older GPs intermediate, and older BPs the worst performers (Fig. 1d,e). In contrast, although the performance in the behavior flexibility task was significantly different between age categories (only for the time spent in the new quadrant) and performance groups, performance of younger and older GPs was similar, as was that of younger and older BPs (Tukey’s test p > 0.05), without a significant interaction between age and performance group (Table 1, Fig. 1f).

**Table 1:** Results of repeated measures, *t*-test and two-way ANOVA on the data obtained from younger and older animals.

Repeated measures	**Behavioral assessment** (Fig. 1d–f)												
	**Working Memory** (**number of GPs**, **BPs**)												
		***df***	***F***		***P***	***ηρ*** ^**2**^							
	Older animals (GPs n = 89, BPs n = 87)	1,174	**236**.**373**		**<0**.**0005**	**0**.**576**							
	Younger animals (GPs n = 63, BPs n = 39)	1,100	**97**.**730**		**<0**.**0005**	**0**.**494**							
	**Reference Memory** (**number of GPs**, **BPs**)												
	Older animals (GPs n = 99, BPs n = 77)	1,174	**181**.**672**		**<0**.**0005**	**0**.**511**							
	Younger animals (GPs n = 61, BPs n = 41)	1,100	**79**.**790**		**<0**.**0005**	**0**.**444**							
t-test	**Behavioral Flexibility - New Quadrant** (**number of GPs**, **BPs**)												
		***df***	***t***	***P***			***d***						
	Older animals (GPs n = 62, BPs n = 114)	**174**	**−18**.**886**	**<0**.**0005**			**2**.**882**						
	Younger animals (GPs n = 40, BPs n = 61)	**99**	**13**.**685**	**<0**.**0005**			**2**.**738**						
	**Behavioral Flexibility - Old Quadrant** (**number of GP**, **BP**)												
	Older animals (GPs n = 62, BPs n = 114)	**158**	**5**.**047**	**<0**.**0005**			**0**.**761**						
	Younger animals (GPs n = 40, BPs n = 61)	**99**	**−5**.**217**	**<0**.**0005**			**1**.**063**						
Two-way ANOVA	**Behavioral assessment** (Fig. 1d–f)		**Performance**					**Age**			**Interaction**		
		***df***	***F***		***P***	***ηρ*** ^**2**^		***F***	***P***	***ηρ*** ^**2**^	***F***	***P***	***ηρ*** ^**2**^
	Working Memory	1,274	**273**.**651**		**<0**.**0005**	**0**.**500**		**245**.**073**	**<0**.**0005**	**0**.**472**	**10**.**753**	**0**.**001**	**0**.**038**
	Reference Memory	1,274	**213**.**618**		**<0**.**0005**	**0**.**438**		**238**.**943**	**<0**.**0005**	**0**.**466**	**10**.**141**	**0**.**002**	**0**.**036**
	**Behavioral Flexibility**												
	New quadrant	1,273	**499**.**625**		**<0**.**0005**	**0**.**647**		**6**.**291**	**0**.**013**	**0**.**023**	0.823	0.365	0.003
	Old quadrant	1,273	**42**.**565**		**<0**.**0005**	**0**.**135**		0.738	0.391	0.003	0.153	0.696	0.001
	**Morphological analysis - Hippocampus** (Fig. 2e,i,m)												
	Granular Neurons	1,35	3.276		0.079	0.086		**67**.**480**	**<0**.**0005**	**0**.**658**	**22**.**030**	**<0**.**0005**	**0**.**386**
	CA3 pyramidal neurons (apical tree)	1,36	2.513		0.112	0.065		**4**.**784**	**0**.**035**	**0**.**117**	0.183	0.671	0.005
	CA3 pyramidal neurons (basal tree)	1,36	2.586		0.116	0.067		**11**.**326**	**0**.**002**	**0**.**239**	0.763	0.388	0.021
	CA1 pyramidal neurons (apical tree)	1,38	2.242		0.143	0.056		**29**.**918**	**<0**.**0005**	**0**.**441**	**9**.**781**	**0**.**003**	**0**.**205**
	CA1 pyramidal neurons (basal tree)	1,38	3.356		0.075	0.081		2.045	0.161	0.051	0.554	0.461	0.014
	**Sholl analysis - Hippocampus** (Fig. 2f,j,n)												
	Granular Neurons	1,35	3.482		0.070	0.090		**44**.**316**	**<0**.**0005**	**0**.**559**	**13**.**869**	**0**.**001**	**0**.**284**
	CA3 pyramidal neurons (apical tree)	1,36	3.022		0.091	0.077		2.254	0.142	0.059	1.049	0.313	0.028
	CA1 pyramidal neurons (apical tree)	1,38	0.059		0.809	0.002		3.546	0.067	0.085	2.456	0.125	0.061
	**Western Blot Data - Hippocampus** (Fig. 3a,b,d–g)												
	LC3-II	1,30	**9**.**964**		**0**.**004**	**0**.**270**		1.112	0.299	0.040	0.270	0.608	0.010
	P62	1,30	1.101		0.303	0.039		1.545	0.225	0.054	1.930	0.176	0.067
	BDNF	1,30	0.526		0.475	0.019		0.128	0.724	0.005	2.126	0.156	0.073
	PSD95	1,30	1.876		0.182	0.065		2.066	0.162	0.071	1.175	0.288	0.042
	Synaptophysin	1,29	1.626		0.214	0.059		0.012	0.913	0.000	2.558	0.122	0.090
	SNAP25	1,30	0.595		0.447	0.022		2.202	0.149	0.075	**4**.**944**	**0**.**035**	**0**.**155**

A subsequent analysis of each individual’s cluster membership for each of the behavioral tests revealed that these were poorly correlated, as animals were distributed across all possible combinations of GPs and BPs, without a clear predominance of impaired cognitive performance across all tests. Importantly, although cognitive performance patterns were widely variable between individuals of the same age group, for each age group, the same eight different patterns of performance were identified, and their overall distribution was approximately similar across the different ages (Fig. 1g). Of notice, the percentage of animals belonging to the GPs group in all the tests was lower in older animals when compared with younger animals (older = 13%, younger = 19%), while the proportion of animals belonging to BPs in all the tests was similar between the different age groups (older = 22%, younger = 19%).

### Age triggers dendritic atrophy that correlates with individual cognitive performance

**Figure 2:**
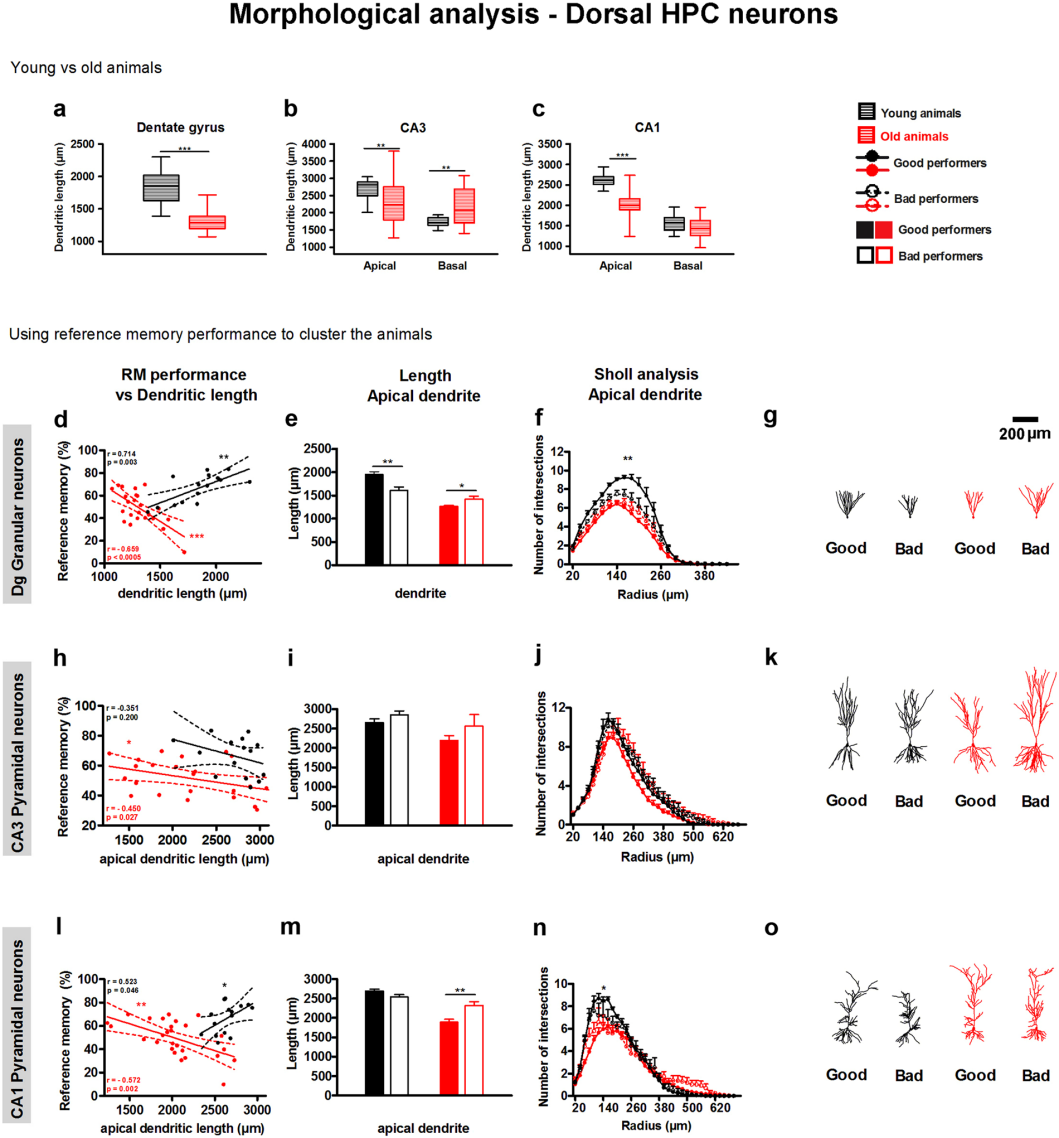
Morphological analysis of HPC neuron dendritic arborizations. When a random sample of all animals is considered (older = 27; younger = 15). (**a**–**c**) Comparison of dendritic lengths of DG granular, CA3 and CA1 pyramidal neurons between younger and older rats. (**d**) Correlation between granular neuron dendritic lengths and individual performances in the reference memory task of both younger and older rats. When similar age animals are clustered (see methods for details) in GPs and BPs according to reference memory performance (older GPs = 16 (18 for CA1); older BPs = 8 (9 for CA1); younger GPs = 10; younger BPs = 5). (**e**) Average dendritic lengths for both GPs and BPs of younger and older animals. (**f**) Sholl analysis of the apical dendrite of DG granular neurons. This graph presents the mean number of intersections of apical dendritic branches with consecutive 20 µm spaced concentric spheres. (**g**) Representative reconstructions of DG granular neurons used in the previous analysis. (**h**–**k**) The same analysis was performed for CA3 pyramidal neurons and in (**l**–**o**) for CA1 pyramidal neurons. Error bars represent SEM, doted lines represent confidence intervals and continuous lines are linear fits; *p < 0.05; **p < 0.01; ***p < 0.001. (RM – reference memory).

To better understand the above described behavioral differences, we analyzed the morphology of dorsal HPC neurons (dentate gyrus (DG) granular, cornus ammonis (CA) 3 and CA1 pyramidal neurons). Considering the granular neurons of the HPC, older animals presented shorter dendritic trees when compared to younger animals (*t*(37) = −8.314, *p* < 0.0005, *d* = 2.736, Fig. 2a). Regarding CA3 and CA1 HPC pyramidal neurons, apical dendrites presented an age-dependent decrease in dendritic length whereas basal dendrites presented no differences in CA1 neurons but an increased dendritic length in CA3 pyramidal neurons (CA3 apical dendrite *t*(37) = −2.907, *p* = 0.006, *d* = 0.807; CA3 basal dendrite *t*(28) = 3.968, *p* = 0.0004, *d* = 1.027; CA1 apical dendrite *t*(38) = −7.282, *p* < 0.0005, *d* = 1.914; CA1 basal dendrite *t*(40) = −1.714, *p* = 0.094, *d* = 0.552; Fig. 2b,c).

Morphological analysis of HPC neurons, in both young and older animals, revealed a correlation between dendritic length and the individual performances in the reference memory task. Curiously, while for younger animals the apical dendritic trees of DG and CA1 neurons presented a significant positive correlation (DG: r^2^ = 0.714, p = 0.003; CA1: r^2^ = 0.523, p = 0.046), in older animals this correlation was reversed, with better performers having the smallest apical trees in all three regions analyzed (DG: r^2^ = −0.659, p < 0.0005; CA3: r^2^ = −0.450, p = 0.027; CA1: r^2^ = −0.572, p = 0.002) (Fig. 2d,h,l). We found a similar negative correlation, in older, but not younger animals, between working memory performance and dendritic tree length of DG, CA3 (apical) and CA1 (apical and basal) cells (Supplementary Table S2). Significantly, there was no correlation in older animals between behavioral flexibility performance (a more mPFC-dependent task) and dendritic length of any HPC cell type (Supplementary Table S2; for additional information regarding individual animal performance in all cognitive tasks see Supplementary Fig. S3a–c).

To further clarify the relationship between HPC dendritic length, age and reference memory performance, we conducted a two-way ANOVA using the clustering of animals according to performance on this test. This analysis revealed a significant effect of age, but not of performance group, and a significant interaction between the two factors, in the average dendritic length of granular neurons and the apical tree of CA1 pyramidal cells (Table 1, Fig. 2e,m). In contrast, no factor seemed to influence the length of CA1 pyramidal cell basal dendrites (Table 1). Regarding comparisons within age groups, old BPs presented a significant increase in the dendritic length of both granular and apical dendrite of CA1 pyramidal neurons when compared with old GPs (DG: *t*(22) = −2.632, *p* = 0.015, *d* = 1.033; CA1 apical dendrite: *t*(25) = −3.312, *p* = 0.003, *d* = 1.382) (Fig. 2e,m). Also, regarding granular neurons, a significant difference was observed between young GPs and BPs. Here, GPs display higher dendritic lengths when compared with BPs (*t*(13) = 3.540, *p* = 0.004, *d* = 1.952) (Fig. 2e). Data on CA3 pyramidal neurons revealed a significant effect of age, but not of performance group nor any interaction in the length of both basal and apical dendrites (Table 1, Fig. 2i).

To explore in which parts of the dendritic tree laid the above-mentioned differences, we performed a Sholl analysis, which measures the number of intersections as a function of distance from the soma. Results for granular dendrites revealed a significant effect of age, but not of performance group, and a significant interaction between the two (Table 1, Fig. 2f). Repeated measures ANOVA revealed that younger GPs, when compared with the BP group, had an overall increase in the number of intersections (*F*_(1,13)_ = 11.050, *p* = 0.005, ηρ² = 0.459), both proximally and distally (Fig. 2f). Results of the Two-way ANOVA analysis for CA3 and CA1 apical dendrites revealed no significant effect of age, performance, neither an interaction between these two factors (Fig. 2j,n). However, group comparisons revealed an overall increase in the number of intersections in apical CA1 dendrites of young GPs when compared with the BP group (*F*_(1,13)_ = 6.294, *p* = 0.026, ηρ² = 0.326) (Fig. 2n). These alterations observed in HPC neurons are exemplified in the reconstructions of Fig. 2g,k,o.

Since some of the cognitive tasks assessed in this work were mPFC-dependent, we also analyzed the morphology of mPFC neurons (cingulate/prelimbic (Cg/PL) and infralimbic (IL) pyramidal neurons; Supplementary Fig. S4). We found an age-dependent reduction in the length of basal dendrites of Cg/PL pyramidal neurons, but no major changes in other dendritic domains (Supplementary Fig. S4a,b). Pearson correlations between behavior performance and dendritic length showed a significant association between working memory and the apical dendritic length of Cg/PL, that was positive in younger subjects (r^2^ = 0.630, p = 0.012) and negative in older animals (r^2^ = −0.504, p = 0.007) (Supplementary Fig. S4c). The performance in the reference memory task was only significantly negatively correlated with the apical dendritic length of IL pyramidal neurons of older animals (r^2^ = −0.465, p = 0.029) (Supplementary Fig. S4o). Regarding the behavioral flexibility task, only in younger animals a positive correlation was found between this task and the apical dendritic length of IL pyramidal neurons (r = 0.611, p = 0.046; Supplementary Table S5; for additional information regarding individual animal performance in all cognitive tasks see Supplementary Fig. S3e,f). In addition to individual correlations, we performed within group comparisons (older and younger GPs and BPs for each task) on average dendritic lengths and distribution of dendritic processes. Interestingly, differences were only present between IL neurons of GPs and BPs in younger animals (*t*(10) = 2.377, *p* = 0.039, *d* = 1.550) (Supplementary Fig. S4p). Finally, the distribution of dendritic processes resulting from Sholl analysis in mPFC neurons showed a significantly more ramified apical dendritic tree of IL pyramidal neurons in younger GPs as compared with younger BPs (*F*_(1,10)_ = 9.339, *p* = 0.012, ηρ² = 0.483), with no differences in the other parameters (Supplementary Fig. S4q). For a comprehensive overview of the simultaneous alterations occurring at different brain regions, Supplementary Fig. S6 depicts the individual morphological alterations of the analyzed animals, including animal performance and respective relative dendritic lengths of DG, CA3, CA1, Cg-PL and IL brain regions.

### Impaired autophagy impacts on dendritic structure

**Figure 3:**
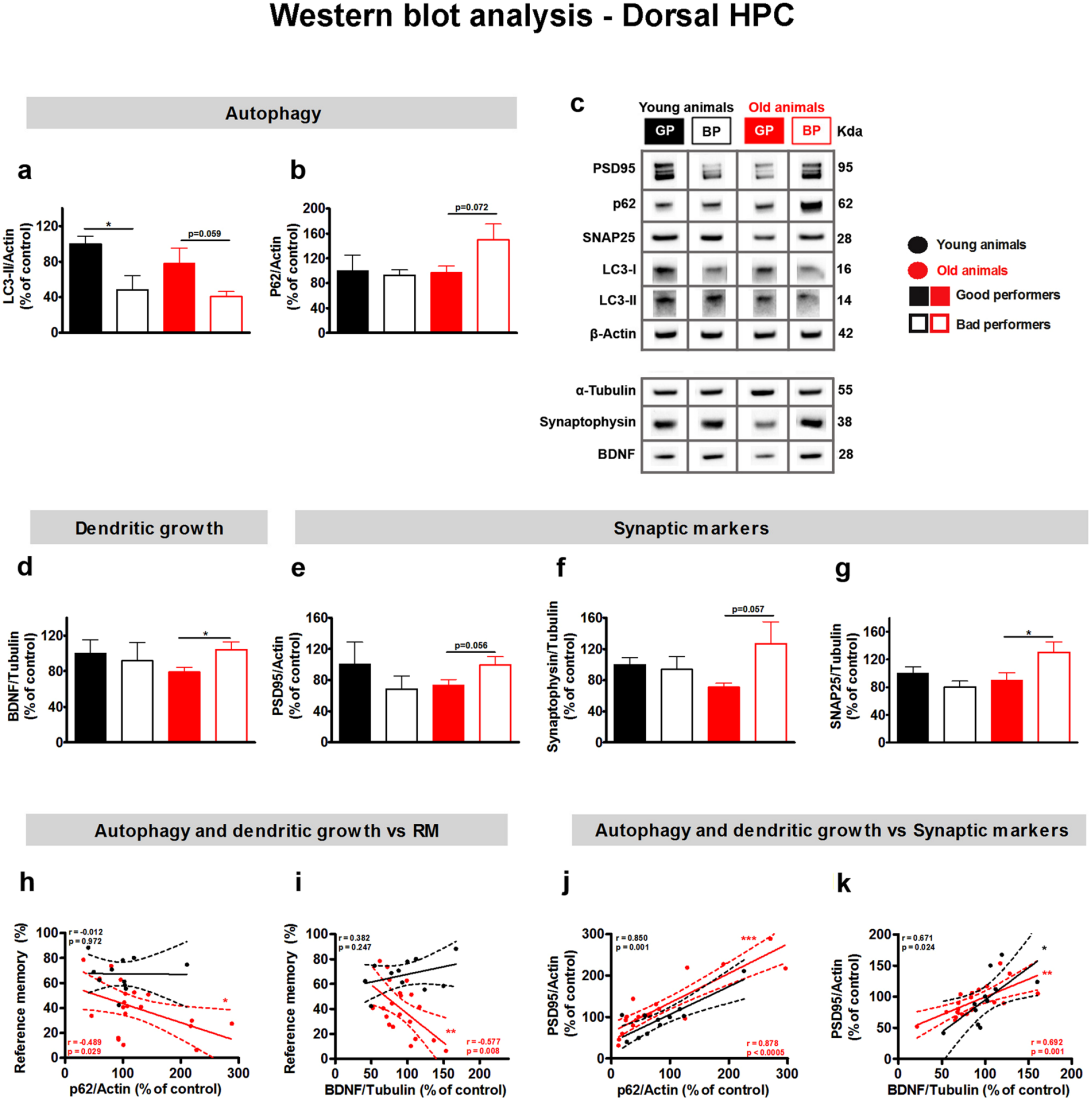
Defective autophagy signaling and dendritic pruning in the HPC of older BPs. Performance in reference memory was used to cluster (see methods for details) both younger and older animals as GPs and BPs. A random sample of these were used for molecular analyses (younger GPs = 5–6; younger BPs = 5; older GPs = 10; older BPs = 9–10). (**a**,**b**) Levels of autophagy markers, LC3-II (**a**) and p62 (**b**), normalized to actin. (**d**) BDNF levels normalized to tubulin. (**e**–**g**) Levels of synaptic markers PSD95, SYP, and SNAP25 normalized to actin, tubulin, and tubulin, respectively. (**c**) Representative western blots of PSD95, p62, SNAP25, LC3, Actin, Tubulin, SYP, and BDNF. For each protein, the blots were cropped from different parts of the same gel. (**h**,**i**) Correlation between RM performance and p62 or BDNF levels, respectively. (**j**,**k**) Correlation between PSD95 and p62 or BDNF levels, suggesting a relationship between the levels of synaptic markers and autophagy or dendritic growth, respectively. Error bars represent SEM, doted lines represent confidence intervals and continuous lines are linear fits; *p < 0.05; **p < 0.01; ***p < 0.001. (RM – reference memory).

Autophagic activity has been identified as a critical mechanism underlying dendritic remodeling^25^. To test the hypothesis that autophagy signaling is disrupted in aging and could be associated with alterations in dendritic recycling in the HPC, we performed western blot analysis of the autophagosome markers: microtubule-associated protein 1A/1B-light chain 3 (LC3) and nucleoporin 62 (p62). To determine the relationship between autophagic activity and dendritic size, protein levels of BDNF were analyzed. As for structural markers of synaptic function, the levels of postsynaptic density protein 95 (PSD95), synaptosomal-associated protein 25 (SNAP25) and synaptophysin (SYP) were determined (Fig. 3c).

To test the association between dendritic length and autophagy/neurotrophin levels, the levels of the synaptic marker PSD95 (a surrogate marker of dendritic extension) was correlated with both p62 (whose increased levels represent decreased autophagic activity) and BDNF. In both younger and older animals, HPC levels of PSD95 were positively correlated with p62 (younger: *r* = 0.850, *p* = 0.001; older *r* = 0.878, *p* < 0.0005; Fig. 3j) and BDNF (younger: *r* = 0.671, *p* = 0.024; older *r* = 0.692, *p* = 0.001; Fig. 3k).

Two-way ANOVA revealed a significant effect of reference memory performance, but not of age, neither an interaction between them, in the HPC levels of LC3-II, but not of p62 (Fig. 3a,b; Table 1). Group comparisons further revealed that, in younger animals, the levels of LC3-II in the HPC were significantly lower in BPs than in GPs (*t*(9) = 2.988, *p* = 0.015, *d* = 1.750; Fig. 3a), while the levels of p62 in the HPC were similar between the two groups. In older animals, there was a trend toward BP animals having less HPC LC3-II (*t*(18) = 2.020, *p* = 0.059, *d* = 0.903); Fig. 3a) and more p62 (*t*(18) = −1.912, *p* = 0.072, *d* = 0.855; Fig. 3b). At the individual level, younger animals had a significant positive correlation between performance in the reference memory task and the level of HPC LC3-II (*r* = 0.790, *p* = 0.004), but not of p62, while in older rats, there was a significant correlation between reference memory performance and the levels of both HPC LC3-II (*r* = 0.523, *p* = 0.018) and p62 (*r* = −0.489, *p* = 0.029; Fig. 3h) (see also Supplementary Table S7). There were no significant correlations between any HPC autophagy markers and performance in the working memory or behavioral flexibility tasks (Supplementary Table S7).

Hippocampal BDNF protein levels were similar in younger BPs and GPs, but were significantly higher in older BPs than in older GPs (*t*(18) = −2.500, *p* = 0.022, *d* = 1.118; Fig. 3d). In younger animals, there was also a trend toward a positive correlation between HPC BDNF levels and performance in working memory (*r* = 0.610, *p* = 0.061) but not in the reference memory task (*r* = 0.382, *p* = 0.247; Fig. 3i) nor in the behavioral flexibility task (Supplementary Table S7). In older rats, HPC BDNF levels were significantly negatively correlated with the performance in reference and working memory tasks (reference memory: *r* = −0.577, *p* = 0.008; Fig. 3i; working memory: *r* = −0.447, *p* = 0.048) but not the behavioral flexibility task (Supplementary Table S7).

Regarding synaptic markers, only HPC SNAP25 levels (Fig. 3g, Table 1) had a significant interaction between age and performance group, without a significant effect of either factor alone. In addition, HPC levels of PSD95 (Fig. 3e), SYP (Fig. 3f) and SNAP25 (Fig. 3g) were different between GPs and BPs solely in older animals. In line with data from BDNF, a significant increase was observed in the levels of SNAP25 and a trend towards an increase of PSD95 and SYP in older BPs, when compared with older GPs (SNAP25 *t*(18) = −2.192, *p* = 0.042, *d* = 0.980; PSD95 *t*(18) = −2.045, *p* = 0.056, *d* = 0.914; SYP *t*(17) = −2.042, *p* = 0.057, *d* = 0.912). Lastly, concerning older animals, HPC PSD95 levels showed a significant negative correlation with reference (*r* = −0.448, *p* = 0.048) and working memory (*r* = −0.513, *p* = 0.021) performances, while HPC SNAP25 levels presented solely a significant negative correlation with the performance in the reference memory task (reference memory task: *r* = −0.560, *p* = 0.010, working memory task: *r* = −0.339, *p* = 0.143). For HPC SYP levels, a trend toward a negative correlation with reference memory (*r* = −0.453, *p* = 0.052) and working memory tests (*r* = −0.406, *p* = 0.085) was observed. No correlations were found between synaptic markers and the performance in the behavioral flexibility task (Supplementary Table S7; for additional information regarding individual animal performance in all cognitive tasks see Supplementary Fig. S3d).

As previously described for the HPC, we performed an analysis of autophagic- and dendritic growth-related proteins in the mPFC (Supplementary Fig. S8). When animals were grouped by performance in working memory, within group analysis revealed that in younger animals, levels of LC3-II and p62 were similar between GPs and BPs whereas in older BPs there was a significant decrease in the levels of LC3-II (*t*(16) = 2.197, *p* = 0.043, *d* = 1.045) and a significant increase in the levels of p62 (*t*(17) = −3.326, *p* = 0.004, *d* = 1.515), suggesting a lower level of autophagy in older BPs (Supplementary Fig. S8a,b). Also, older animals, but not younger animals, presented a negative correlation between p62 and the performance in reference (*r* = −0.504, *p* = 0.028) and working memory tasks (*r* = −0.646, *p* = 0.003; Supplementary Fig. S8h), but not the behavioral flexibility task (Supplementary Table S9). Regarding neurotrophins and synaptic markers, mPFC BDNF, PSD95 and SYP protein levels were significantly higher in older BPs compared with older GPs (BDNF *t*(18) = −2.226, *p* = 0.039, *d* = 0.995; PSD95 *t*(16) = −1.959, *p* = 0.068, *d* = 0.937; SYP *t*(18) = −2.108, *p* = 0.049, *d* = 0.943) (Supplementary Fig. S8d–f). Also, BDNF levels were not correlated with performance in working memory (Supplementary Fig. S8i), reference memory or behavioral flexibility (Supplementary Table S9) in any age group, and were correlated with PSD95 levels in older animals (*r* = 0.671, *p* = 0.002; Supplementary Fig. S8k) but not younger rats (*r* = −0.256, *p* = 0.476; Supplementary Fig. S8k; for additional information regarding individual animal performance in all cognitive tasks see Supplementary Fig. S3g). This data is in agreement to what we previously described for the HPC, further showing that the levels of autophagy in mPFC neurons are strongly and positively related with dendritic pruning and better performances in older individuals.

## Discussion

The present study addressed the heterogeneity of cognitive aging from a multidimensional perspective to reveal a hitherto of unappreciated complexity. It explored its underpinnings, highlighting, for the first time, the role of the balance between neurotrophic and autophagic activities in such processes. Data herein presented confirmed that, despite a general aging-associated cognitive decline, the performance of both young adult and older rats in working and reference memory tasks is heterogeneous, particularly in older individuals^27^; in the latter, we confirmed that a certain proportion of subjects maintain spatial memory abilities comparable to those of younger animals^6,7^. Of notice, the data clearly revealed, for the first time, that this age-associated increase in the dispersion of individual performance is not universal, as it was not present in all cognitive dimensions (e.g. the behavioral flexibility task).

The working and reference memory tasks both assess spatial learning and memory, albeit within a different timeframe: while the former is dependent on short-term memory and HPC to mPFC connections^28,29^, the latter depends on long-term memory and largely on the integrity of the HPC^30^. In contrast, the behavioral flexibility task is memory independent and assesses the ability to adapt to changing circumstances^31^, a critical component of executive function, which is a very distinct cognitive ability. In light of this distinction, the observation that aging is accompanied by an increased inter-individual heterogeneity (and a performance decline, on average) in memory-dependent but not executive-function-dependent tasks adds yet another layer of heterogeneity to the aging process, and strongly suggests that some cognitive functions (and the networks sub-serving them) are more prone to aging than others. Significantly, this seems also to be the case in humans^32^ in which both long-term and working memory are more influenced by age-related impairments than knowledge of vocabulary and priming, a form of non-declarative memory. Despite this, the herein reported relative preservation of executive function in rodents might seem to contradict several studies in humans showing that executive processes are also disrupted in aging^33–35^. However, it is important to highlight that, besides the obvious species difference, executive function tasks in humans are often contaminated by deficits in speed of processing^36^, which are well known to be affected by aging. On the contrary, in our experiments, not only was average swimming speed similar in all older subjects (and not significantly different from that of their younger counterparts) but also the behavioral flexibility test is independent of it.

In addition to the two layers of heterogeneity in aging discussed above, analysis of each animal’s membership to either the GPs or BPs group, for each behavioral test, further revealed another dimension of inter-individual heterogeneity. Indeed, when comparing cluster membership for each individual in each test, we showed for the first time that most animals were GPs in some tests and BPs in others, without a clear separating pattern in either younger or older groups. Moreover, the overall distribution of animals according to their group membership (GPs or BPs) for the 3 tests (working and reference memory and behavioral flexibility) was strikingly similar in both age groups, with only 13% of older and 19% of younger animals being good in every task, and 22% of older and 19% of younger animals being bad in every task. More importantly, this third heterogeneity level seems to be independent of the other two. In other words, despite all the inter-individual heterogeneity (within a given test) and the heterogeneity between tests (with reference and working memory being more sensitive to aging than behavioral flexibility), there is also heterogeneity at the individual level, as to being a GP or BP for each behavioral test.

In summary, the behavioral data suggest that cognitive decline in aging is not inevitable, or strictly linked to chronological age and that, even in a relatively homogeneous population of animals such as the one in this study, there is a high variability and complexity in the way the different cognitive functions are preserved/impaired in each individual. Understanding the underpinnings of individual differences may help to explain the observed heterogeneity and, possibly, what determines the existence of healthier agers, which was next studied at the morphological and molecular levels.

Changes in the morphology of neuronal dendritic trees were shown to correlate with cognitive alterations in both rodents and humans^37,38^. In aging, it is already well established that memory impairments are not related with neuronal loss^27,39^, but rather to volume changes^17^ and altered morphology of neuronal dendritic trees^40,41^. In the present work, using a large number of rats (15 young and 27 old) we showed that, on average, older animals have shorter apical dendritic arborizations in dorsal HPC neurons (DG granules and CA1 and CA3 pyramids) but similar apical dendritic trees in mPFC neurons (Cg/PL and IL layers II/III pyramids) when compared to younger animals. These findings are in line with most previous studies that analyzed one or the other region (HPC^42–47^; mPFC^48–50^) and might suggest that the frontal regions might be less affected by the aging process or that age-related changes in neuronal morphology appear later in the mPFC. This is partly corroborated by the fact that age-related apical dendritic retraction in the mPFC was only reported in one study^51^. Interestingly, this relative mPFC “resilience” might be specific for the superficial layers, since deeper layer V pyramidal neurons, similar to hippocampal cells, exhibit an age-related apical dendritic retraction at 20–22 months^20,47,50^. Importantly, this has been observed in humans in which age-related dendritic retraction was 3 times more prominent in the deep than in the superficial PFC pyramids^52^. The fact that mPFC dendrites are less affected by aging fits perfectly with the behavioral data presented here, pointing to an attenuated age-associated decline of executive functions.

Another major novelty of the present work is the finding that older animals with deficits in HPC-dependent tasks have larger dendritic trees in the HPC than cognitively intact rats of the same age. Some previous papers had already shown that HPC cells from older rats^53^ and older humans^54,55^ had increased dendritic length, but in none of these studies were subjects cognitively characterized. Interestingly, in the mPFC, despite no overall age-related retraction, there was a similar, albeit with smaller magnitude, association between bigger apical dendritic trees in layer II/III Cg/PL pyramids and worse performance in the working memory (mPFC-dependent) test. This association, in older animals, between larger dendritic trees and poorer cognitive function might be considered contra-intuitive. However, while bigger dendritic trees might mean more connectivity and better neuronal function, it is also well described that the accumulation of “waste” dendritic material and large dendritic trees, for example as a result of impaired autophagy and dendritic pruning deficits, hampers neuronal function and correlates with cognitive deficits, in both humans and animals^23^. Interestingly, in Fragile X syndrome patients, who have impaired dendritic pruning, there is also an inverse correlation between HPC volume and cognitive performance, which is not present in age-matched individuals without pruning deficits^56^. Of note, in the present work, the inverse correlations between dendritic tree length and cognitive performance are not present in the group of younger adults (in which an opposite trend is observed), supporting an age-related phenomenon. Together, these results suggest that the inverse correlation between large dendritic trees and poor cognitive performance in the elderly might be attributed to age-associated dendritic pruning deficits, leading to larger, less efficient, dendritic trees. In light of this hypothesis, individual differences in dendritic pruning might also underpin the individual heterogeneity in cognitive aging.

Dendritic pruning is a mechanism often used to selectively remove unnecessary and exuberant neuronal branches, not only in the immature nervous system^23^, but also in the adult HPC^57^, thus ensuring the proper formation of functional optimized circuitries. Dendritic and synaptic pruning is highly dependent on autophagy-dependent protein turnover, as animals presenting constitutional^23^ or induced^58^ inhibition of autophagy have larger dendritic trees and increased spine density, which correlate with cognitive deficits. In order to further dissect whether this could contribute to the observed morphology, we analyzed the levels of autophagic activity in the HPC and mPFC. Furthermore, this was complemented with a quantification of the neurotrophin BDNF, a main inducer of dendritic and spine growth^59^. Finally, given the technical challenge to assess protein levels and dendritic tree length in the same region of the same animals, these results were correlated with pre- and post-synaptic markers. Indeed, there is a consensus in the literature that these levels, particularly when concordant, are a good surrogate of synaptic abundance and dendritic tree complexity^60–62^. In support of this assumption, here we show that older, but not young, cognitively impaired animals have higher levels of these synaptic proteins than age-matched cognitively intact rats, precisely replicating the findings from the dendritic tree analysis.

With the present work, we reveal that older cognitively impaired animals have reduced autophagic activity in both the dorsal HPC and the mPFC, when compared with older cognitively intact rats. Of notice is the fact that a decrease in the relative abundance of the autophagic vacuole marker LC3-II (lipidated LC3) was accompanied by a correspondent increase in the relative abundance of the autophagy cargo-protein p62^63^, attesting the robustness of the findings. Significantly, these observations were specific for older animals, as levels of autophagic markers in both brain regions did not differ between cognitively intact and cognitively impaired younger individuals. In most organisms, pathological aging is associated with decreased autophagic activity and autophagy inhibition induces degenerative changes that resemble those associated with aging^64^. While the mechanisms of such relationship are far from being well understood, the most prevalent hypothesis considers a failure to clean toxic/waste protein debris, that accumulate with time and induce cellular dysfunction^64^. In line with this, we found that, in the older, decreased levels of autophagy are strongly and inversely correlated with the abundance of synaptic markers, a surrogate marker of dendritic length. Our findings, however, further extend the interpretation of the previous observations, by suggesting that in neurons, decreased autophagy results in less dendritic pruning and an accumulation of dendrites that hamper neuronal function. Significantly, this does not seem to be an inevitable consequence of aging, as levels of autophagic and synaptic markers in older cognitively intact individuals were strikingly similar to those of younger animals. Of note, BDNF levels similarly did not vary significantly with aging but were also increased in older cognitively impaired, compared with cognitively intact animals, suggesting that increased dendritic growth, as well as decreased autophagic activity, might also contribute to the increased dendritic length observed in these animals.

Many factors could induce a decreased autophagic activity, similar to that presented by older cognitively impaired individuals. One of the best candidates is an enhanced activity of the mechanistic target of rapamycin (mTOR, formerly known as mammalian target of rapamycin mTOR) complex, a redox/energy/nutrient sensor that inhibits autophagy and stimulates protein synthesis^65^. Increased mTOR activity (resulting in decreased autophagic activity) has been linked to cognitive dysfunction and learning deficits in a variety of disorders^66,67^, which are also associated with an increase in dendritic spines^68^. More importantly, and in line with our results, lifelong treatment of mice^69,70^ or accelerated senescence rats^71^ with the mTOR inhibitor rapamycin (that is considered an autophagy inducer) improved age-related cognitive dysfunction. Given the above, it is plausible to conclude that an impairment of neuronal autophagic activity could result in a scarcity of pruning mechanisms in aging neural circuits, leading to an accumulation of dendritic material and to the consequent decrease of cognitive performance.

Other factors that are commonly associated with aging could also impact dendritic length, including altered glutamatergic transmission and insulin signaling. However, since these would ultimately lead to changes in neurotrophins and/or autophagic processes, we did not address these separately in the present work. Nevertheless, in order to gain full insight into the individual determinants of altered autophagic activity, these and other factors should be taken in consideration. Furthermore, insights into the relevance of all these mechanisms can only be obtained by experimental manipulations of autophagy, which were not the scope of the present work but should be pursued in the future.

**Figure 4:**
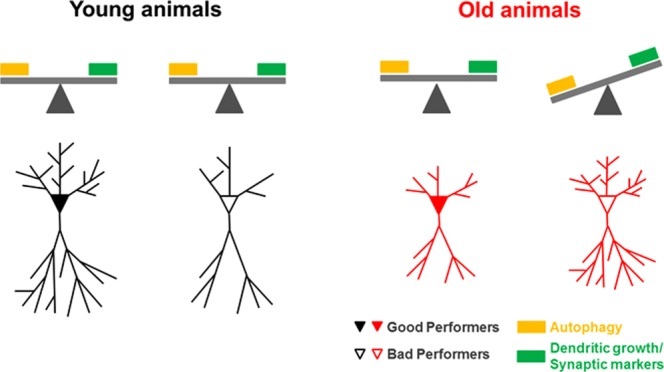
Schematic representation of the relations between age, cognitive performance, neuronal morphology, autophagy, synaptic and dendritic growth markers, in the HPC and mPFC. In younger animals “bigger is better”; GPs have the biggest dendritic trees. However, there are no extensive differences in autophagy levels (LC3-II, p62), dendritic growth (BDNF) and synaptic markers (PSD95, SNAP25, SYP). In older animals, it seems that “smaller is better”. BPs have the bigger dendritic trees, associated with a decrease in the levels of autophagy (LC3-II, p62), and an increase in dendritic growth (BDNF) and synaptic markers (PSD95, SNAP25, SYP).

In conclusion, our findings show that alterations in the dendritic length of neurons are associated with the heterogeneity observed in the performance of young and older animals, with a twist. Indeed, it seems that while for younger animals “bigger is better”, for older animals “smaller is definitely better”. Moreover, we herein provide evidence that, in older animals, dendritic length differences and behavioral heterogeneity can be ascribed to variations in neurotrophin levels and, more importantly, autophagic activity. To summarize these associations, we propose a model where the balance between neurotrophins and autophagic activity regulates dendritic growth/pruning thus contributing to the heterogeneity in the cognitive function of younger vs older animals (Fig. 4). This data represents a paradigm shift in understanding the individual differences observed with aging.

## Methods

### Animals

All procedures were carried out in accordance with local regulations (Decreto-Lei no. 113/2013) and European Union Directive 2010/63/EU on animal care and experimentation. Animal facilities and the people directly involved in animal experiments were certified by the Portuguese regulatory entity – DGAV (Direção-Geral de Alimentação e Veterinária). All protocols were approved by the Ethics Committee of the Life and Health Sciences Research Institute (ICVS). All the male Wistar Han rats (Charles River Laboratories, Barcelona, Spain) used in the study were housed in groups of 2 and maintained under standard laboratory conditions: artificial 12 h light/dark cycle (lights on from 08:00 a.m. to 08:00 p.m.); room temperature 22 °C; *ad libitum* access to food and water. A total of 176 old (22–24 month-old) and 102 younger (4–6 month-old) male rats were used in the study. The animals were tested in a battery of water-maze based tests to assess cognition. The brains of a randomly selected subset of both younger and older animals were subjected to morphological (3D neuron reconstruction) analyses and of another randomly selected subset to molecular analyses (western blot). The remainder animals were sacrificed at various time points for several other analyses not included in the present study. All behavioral testing was conducted during the light phase of the daily light cycle.

### Behavioral assessment

The cognitive status of all the animals was assessed based on performance in a series of tasks using the water maze. Animals were tested during 8 days in 3 tests designed to assess different cognitive domains: spatial working memory, reference memory and behavioral flexibility^72^. The apparatus consisted of a large circular black pool (170 cm diameter), filled to a depth of 31 cm with water (at 22 °C), which was divided by imaginary lines in 4 equal-sized quadrants. During the execution of the test, a submerged cylindrical black platform (12 cm diameter, 30 cm high) was hidden below the water surface at the center of one of the quadrants. The room was dimly lit and extrinsic visual clues were glued to the walls surrounding the tank and kept unaltered during the duration of the experiment. Data was collected using a video camera placed above the center of the pool connected to a video-tracking system (Veiwpoint, Champagne au Mont d’Or, France).

#### Working memory task

This task is a variation of the spatial reference memory test^30^ and depends on mPFC function^28^. Its goal is to assess the ability of rats to learn the position of a hidden platform and to keep this information online during 4 consecutive trials. This test consisted of 4 days of acquisition in which the position of the platform was kept constant during each daily trial (with a maximum of 120 s per trial) but was altered to a different quadrant every changing day (such that all four quadrants were used). Thus, the animal cannot know where the platform was hidden on trial 1 of each day. Test sessions begun with rats facing the wall of the maze, being placed at one of the four different starting points (north, east, south or west) which were different every day. A trial was considered complete once the escape platform had been reached by the rat. The time spent to reach the platform was recorded. Animals were then allowed to spend 30 s in the platform, after which they were towel-dried and allowed to rest in a holding cage some s before being returned to the maze. When the escape platform was not reached within 120 s, the experimenter guided the animal to the platform and an escape latency of 120 s was recorded.

#### Reference memory task

This task is a HPC-dependent task^30^ that evaluates the ability of the animal to learn the location of a hidden platform during 4 consecutive days – spatial reference memory. After the working memory procedure (e.g. on days 5–7), the platform remained in the same quadrant as on day 4 and animals were tested for an additional 3 days without changing the location of the hidden platform. The remaining procedures were like those described above.

#### Behavioral flexibility task

This is a mPFC-dependent task and was performed after the reference memory task (day 8). For this test, the escape platform was moved and located in the opposite quadrant of where it had been for the previous 4 days. All the procedures were similar to those described above. For this task, time spent swimming in each quadrant was recorded and analyzed.

### Histological procedures

Two months after the behavioral evaluation, 50 older (22–24 month-old) and 27 younger (4–6 month-old) rats were randomly selected, deeply anesthetized with sodium pentobarbital and perfused with saline for Golgi-Cox staining (older n = 30, younger n = 15) and western blot analysis (older n = 20, younger n = 12).

#### Golgi-cox staining

After perfusion, the brains were removed, immersed in 25 mL of Golgi-Cox solution^73^ (1:1 solution of 5% potassium dichromate and 5% mercury chloride diluted 4:10 with 5% potassium chromate) and kept in the dark at room temperature for 14 days. Brains were then transferred to a 30% sucrose solution. At this moment, brains were stored in the dark at 4 °C from a minimum of 3 days to a maximum of 2 months, before being cut on a vibratome. Coronal sections (200 µm thick) were collected in 6% sucrose and blotted dry onto cleaned, gelatin-coated microscope slides. Subsequently, sections were alkalinized in 18.7% ammonia, developed in Dektol (Kodak), fixed in Kodak Rapid Fix, dehydrated through a graded series of ethanol of increasing concentrations and cleared in xylene before being covered in mounting media (Entellan New) and coverslipped. The slides were stored in the dark and exposed to the air, at room temperature, until being analyzed.

### Structural analysis

To ensure an unbiased analysis, slides were re-coded by the lab technician (not involved in the research) as soon as they were prepared and all neuronal reconstructions were done blind to animal age or performance group. To minimize bias, codes were only broken after all data was collected and entered into the database.

#### Dendritic tree analysis

Dendritic arborizations were analyzed in the DG, CA3 and CA1 regions of the dorsal HPC, and in layer II/III of the Cg/PL and IL areas of the mPFC. The dorsal/ventral HPC division was performed according to Pinto *et al*.^74^ and the identification of layer II/III of the Cg/PL and IL areas was achieved according to Cerqueira *et al*. (2007b)^75^.

The granular neurons of the DG were readily identified based on their round cell bodies, which are located in the stratum granulosum of the suprapyramidal and infrapyramidal blades. Pyramidal neurons from the HPC (CA1 and CA3) or the mPFC (Cg/PL and IL) were readily identified by their characteristic triangular shaped soma. All neurons were chosen for reconstruction based on the criteria described by Uylings *et al*.^76^: (i) full impregnation of the neurons along the entire length of the dendritic tree; (ii) apical dendrite without truncated branches, except on the most superficial layer; (iii) presence of at least 3 primary basal dendritic shafts, each of which branched at least once (when applicable); (iv) relative isolation from neighboring impregnated cells that could interfere with analysis (clear somatic boundaries) (v) no morphological changes attributable to incomplete dendritic impregnation of Golgi-Cox staining. To minimize selection bias, slices containing the region of interest were randomly searched and the first 5–10 neurons fulfilling the above criteria (maximum of 3 neurons per slice) were chosen. For each selected neuron, all branches of the dendritic tree were reconstructed at 600× magnification, using a motorized microscope (Olympus BX51 Microscope with oil-objectives), attached to a camera (QImaging® Retiga-2000R digital camera, Surrey, Canada) and the Neurolucida software (Microbrightfield, VT, USA). A 3-D analysis of the reconstructed neurons was performed using NeuroExplorer software (Microbrightfield). Dendritic morphology was examined by assessing the total dendritic length and the number of dendritic branches. In addition, to assess differences in the arrangement of dendritic material, a 3-D version of a Sholl analysis^77^ was performed. For this, the number of intersections of dendrites with concentric spheres positioned at radial intervals of 20 µm from the soma was recorded.

### Western blot analysis

Rat brain tissue (dorsal HPC and mPFC) was lysed with 1X RIPA buffer supplemented with protease inhibitors (cOmplete Mini EDTA-free, Roche, Basel, Switzerland) and phosphatase inhibitors (phosphatase inhibitor Cocktail 2 and 3, Sigma-Aldrich, St. Louis, Missouri, US). Homogenization was performed with an electric homogenizer, and homogenates were maintained in constant agitation for 2 h at 4 °C. After that, homogenates were centrifuged at 12000 rpm at 4 °C for 20 min and supernatants were collected for western blotting. Protein concentration was determined using the Bradford assay (Bio-Rad, Hercules, CA, USA). Twenty micrograms of total protein were loaded into SDS-Page gels and then transferred to nitrocellulose membranes. The membrane was stained with Ponceau to verify successful transfer. Membranes were incubated with the following primary antibodies: LC3 (1:1000, Cell signaling, Danvers, US), p62 (1:1000, Novus, St. Charles, US), BDNF (1:1000, Abcam, Cambridge, UK), SNAP25 (1:5000, Abcam), SYP (1:10000, Abcam), PSD95 (1:1000, Abcam), α-tubulin (1:5000, Sigma-Aldrich, St. Louis, Missouri, US), and β-actin (1:1000, Ambion, Naugatuck, US), overnight at 4 °C. Antibody affinity was detected by chemiluminescence (ECL Bio-Rad). Band quantification was done using ImageLab 4.1 (Bio-Rad) using α-tubulin or β-actin as the loading control. Full-length images are presented in Supplementary Fig. S10.

### Statistical analysis

All statistical analysis was conducted in the SPSS software package version 19 (IBM corporation, Armonk, New York, US). After confirmation of homogeneity and normality, appropriate statistical tests were applied to the data. To facilitate direct comparisons between different tests, results of all behavioral tests were converted to a 0–100% scale, where 0% indicates worst possible performance (120 s to reach the platform for the working and reference memory tests or no time in target quadrant for the behavioral flexibility task) and 100% indicates best possible performance (0 s to reach the platform or total time in the target quadrant). Also, in order to allow individual correlations between the performance of the animals in the working and reference memory tests, which consist of several testing days, and structural parameters, a performance index (PI) was calculated for each test by employing the following formula: PI = (P1 + (P3 + P4)/2)/2 where Pn represents the average performance of each animal on trial n (for working memory) or day n (for reference memory); importantly, the index value can be directly read as the average performance of each animal per trial/day. Regarding the behavioral flexibility test, the percentage of time spent in the target quadrant (performance index) was used to assess the individual correlations. Clustering of animals in good and bad performance groups was done using the k-means cluster analysis according to their performance index in the working memory, reference memory or behavioral flexibility tasks. The distance between cluster centers of the two desired groups was maximized in an iterative process (maximum number of iterations set to 25). Comparisons between two groups were done using the two-tailed t-test and repeated measures ANOVA. Two-way ANOVA was used to evaluate the impact of age and the effect of group performance in further behavioral, structural and molecular data. For multiple comparisons in the behavioral tasks, one-way or repeated measures ANOVA were used. Differences between groups were then determined by Tukey´s honestly significant difference test post-hoc analysis. Pearson correlations were computed between continuous variables. Measures of effect size (Cohen’s d, Eta-squared or Pearson correlations) are presented whenever appropriate. Note that, regarding the number of younger animals that performed the behavioral flexibility task, only 101 out of the102 animals were included in the analysis; this was due to tracking problems. Results are expressed as group mean ± SEM. Differences were considered significant if *p < 0.05; **p < 0.01; ***p < 0.001.

## Supplementary information

Supplementary information

## Data Availability

The datasets generated during and/or analyzed during the current study are available from the corresponding author on reasonable request.
