# Supplementary material for: Structural and molecular correlates of cognitive aging in the rat
Source: Sci Rep. 2019 Feb 14;9:2005. doi: 10.1038/s41598-019-39645-w (PMC6376121; doi:10.1038/s41598-019-39645-w)
Supplement: Supplementary file 1 — Supplementary information [file 41598_2019_39645_MOESM1_ESM.pdf]

Structural and molecular correlates of cognitive aging in the rat

Cristina Mota, Ricardo Taipa, Sofia Pereira das Neves, Sara Monteiro-Martins, Susana Monteiro, Joana Almeida Palha, Nuno Sousa, João Carlos Sousa and João José Cerqueira

Supplementary Information S1-S8:

Figure S1| Behavioral assessment of younger and older rats.

Table S2| List of correlations between the performance in working memory or behavioral flexibility tasks and the dendritic length of dorsal HPC neurons.

Figure S3| Cognitive performances in the WM, RM and BF tasks of the animals used in the morphological and molecular analysis.

Figure S4| Morphological analysis of neurons in the mPFC.

Table S5| Correlations between the performance in the behavioral flexibility task and the dendritic length of mPFC neurons.

Figure S6| Morphological alterations in the HPC and mPFC of individual young and old animals.

Table S7| List of correlations between dorsal HPC western blot data and the performance in the reference memory, working memory or behavioral flexibility tasks.

Figure S8| Dysregulation in autophagy signaling and dendritic pruning in the mPFC of older animals.

Table S9| Correlations between mPFC western blot data and the performance in the reference memory, working memory or behavioral flexibility tasks.

Figure S10| Full-length western blots for Figure 3c and supplementary Figure S8c.

Behavioral assessment

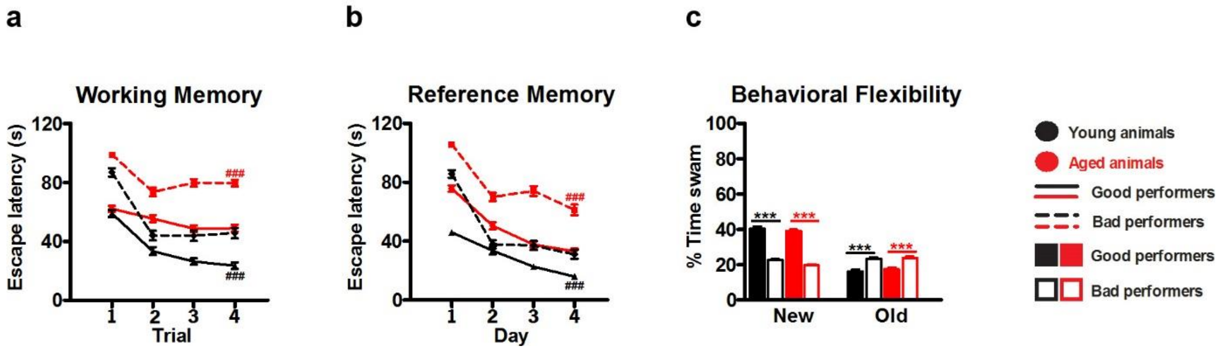

Figure S1| Behavioral assessment of younger and older rats. When similar age animals are clustered (see methods for details) in Good and Bad performers: **A, B)** Learning curves in the working **(A)** and reference memory task **(B)** of GPs and BPs for both younger and older rats. **C)** Results from the behavioral flexibility task. Average time spent on the four trials in each imaginary quadrant is given as a percentage of the total escape latency. Number of animals: working memory - older: GPs n=89, BPs n=87; younger: GPs n=63, BPs n=39; reference memory - older: GPs n=99, BPs n=77; younger: GPs n=61 BPs n=41; behavioral flexibility task - older: GPs n=62 BPs n=114; younger: GPs n=40 BPs n=61. Error bars represent SEM; * p<0.05; *** p<0.001.

Table S2| List of correlations between the performance in working memory or behavioral flexibility tasks and the dendritic length of dorsal HPC neurons.

			Old animals		Young animals	
			**WM**	**BF**	**WM**	**BF**
**Granular neurons**	**Dendritic length**	Pearson Correlation	**-0.458***	0.029	0.159	0.533
		Sig. (2-tailed)	**0.024**	0.894	0.572	0.050
		N	**24**	24	15	14
**CA3 pyramidal neurons**	**Apical dendritic length**	Pearson Correlation	**-0.431***	0.101	-0.230	**-0.649***
		Sig. (2-tailed)	**0.036**	0.645	0.410	**0.012**
		N	**24**	23	15	**14**
	**Basal dendritic length**	Pearson Correlation	-0.123	0.060	-0.173	**-0.585***
		Sig. (2-tailed)	0.567	0.785	0.538	**0.028**
		N	24	23	15	**14**
**CA1 pyramidal neurons**	**Apical dendritic length**	Pearson Correlation	**-0.418***	0.095	0.246	0.351
		Sig. (2-tailed)	**0.030**	0.644	0.377	0.219
		N	**27**	26	15	14
	**Basal dendritic length**	Pearson Correlation	**-0.436***	0.006	0.111	-0.095
		Sig. (2-tailed)	**0.023**	0.976	0.694	0.748
		N	**27**	26	15	14

*p<0.05

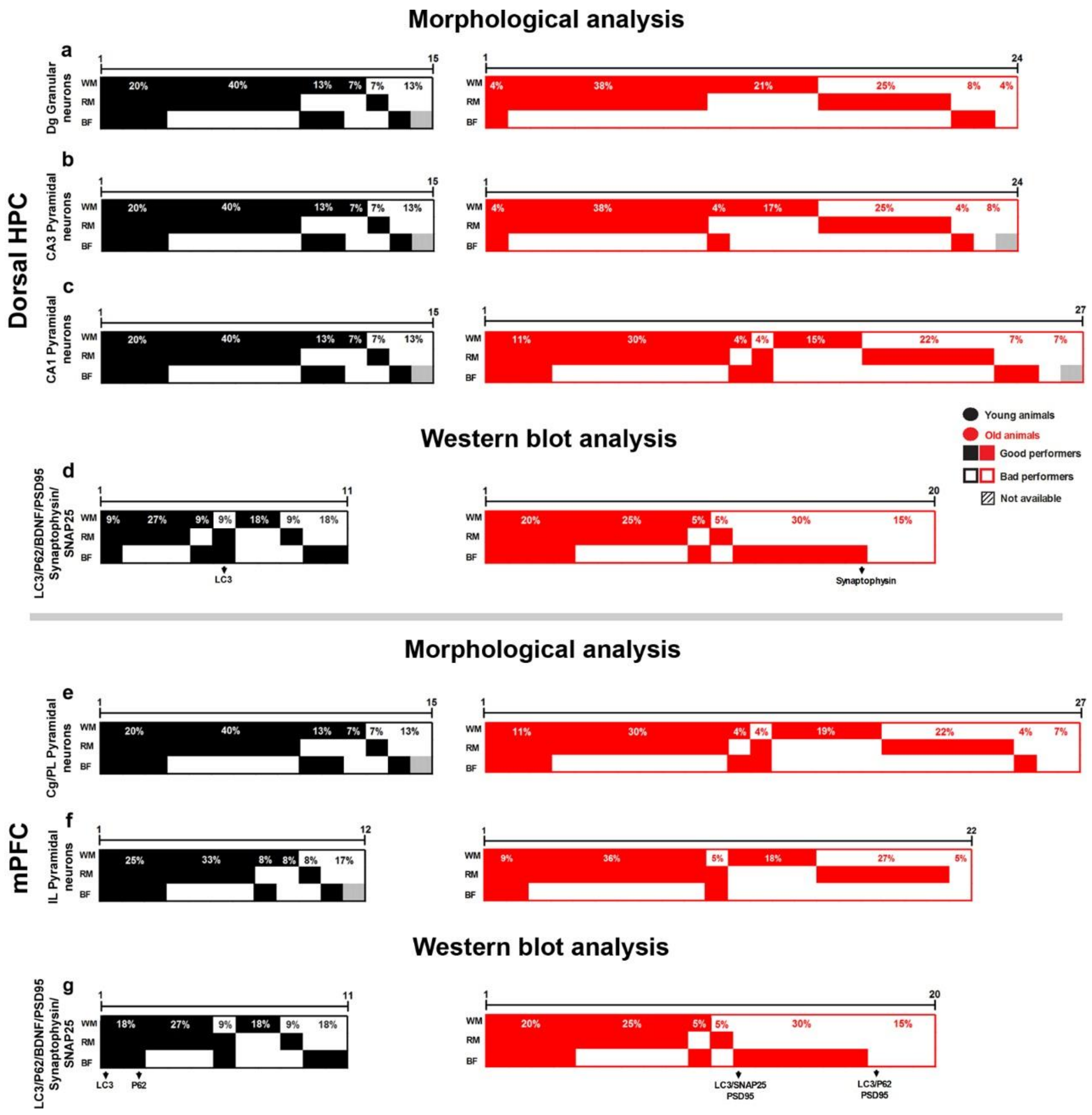

Figure S3| Cognitive performances in the WM, RM and BF tasks of the animals used in the morphological and molecular analysis. A, B, C, E and F) Represent the cognitive cluster of each animal (both young and aged) used for the analysis of the Dg, CA3, CA1, Cg/PL and IL, respectively. **D and G)** Represent the cognitive clusters of each animal (both young and aged) used for HPC **(A)** and mPFC **(B)** western blot analysis of LC3, p62, BDNF, PSD95, SNAP25 and Synaptophysin levels. Arrows indicate missing proteins in the analysis for each animal.

Morphological analysis - mPFC neurons

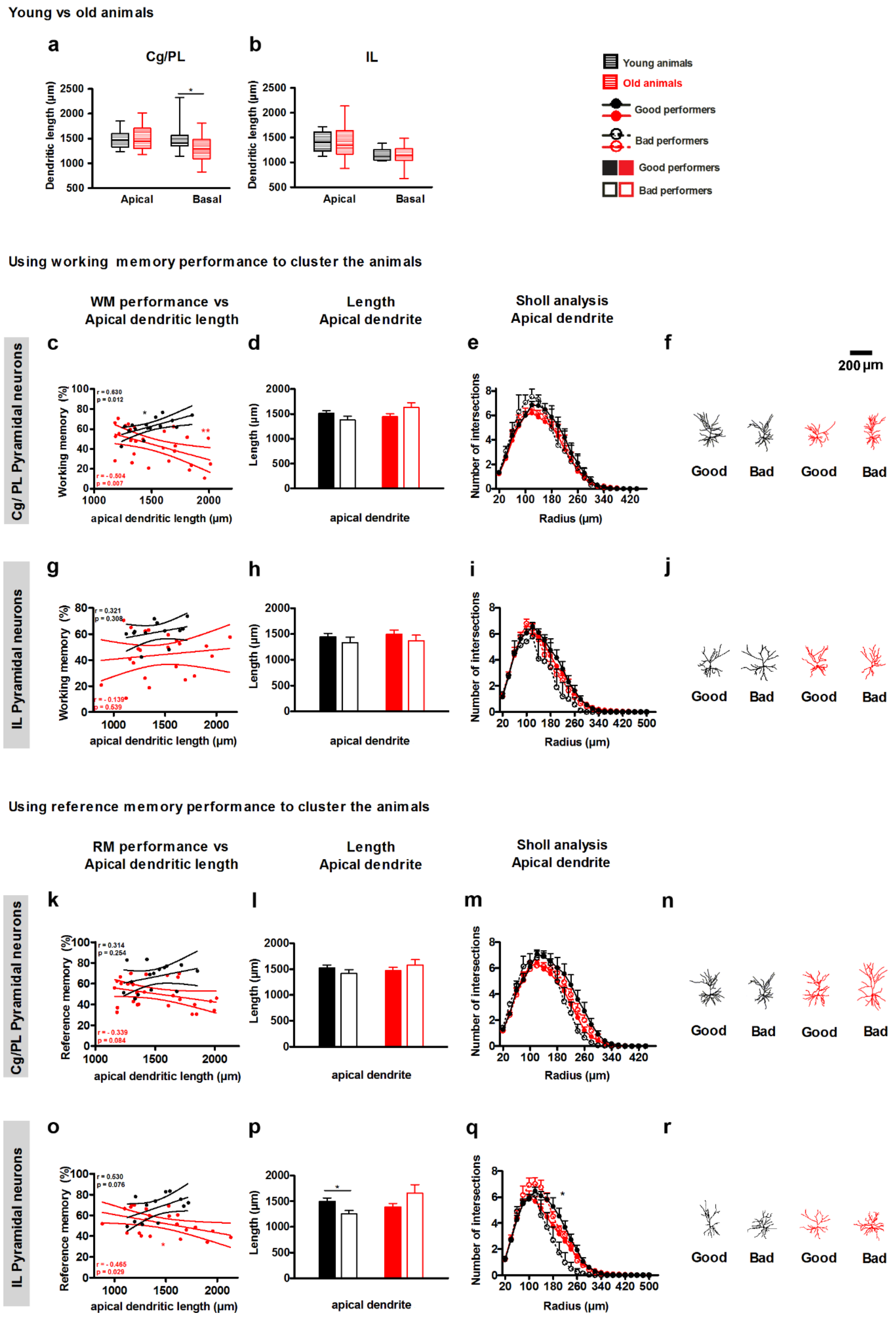

Figure S4| Morphological analysis of neurons in the mPFC. When a random sample of all animals is considered: **A** and **B)** Comparison of dendritic lengths of Cg/PL and IL pyramidal neurons of the mPFC in both older (n=27 for Cg/PL and n=22 for IL neurons) and younger (n=15 for Cg/PL and n=12 for IL neurons) animals. **C** and **K)** Correlation between the individual performances of both younger and older rats in the working and reference memory tasks, respectively, and the apical dendritic length of Cg/PL pyramidal neurons. **G** and **O)** The same correlations were performed for the apical dendritic trees of IL pyramidal neurons. When similar age animals are clustered (see methods for details) in Good and Bad performers according to working or reference memory performance: **D** and **L)** Cg/PL pyramidal neuron apical dendritic lengths using, the performance in the working and reference memory tasks, respectively, to cluster the animals. **H** and **P)** The same procedure was performed for IL pyramidal neurons. **E** and **M)** Sholl analysis of the apical dendrites of Cg/PL pyramidal neurons. **I** and **Q)** The same analysis performed for IL pyramidal neurons. These graphs represent the mean number of intersections of apical dendrite branches with consecutive 20µm spaced concentric spheres. **F**, **J, N** and **K)** Reconstructions of representative Cg/PL and IL pyramidal neurons. Number of animals used for the analysis of the Cg/PL cortex: 15 younger animals (RM cluster GPs=10 and BPs=5; WM cluster GPs=12 and BPs=3) and 27 older animals (RM cluster GPs=18 and BPs=9; WM cluster GPs=17 and BPs=10). For the IL cortex the number of animals used was: 12 younger animals (RM cluster GPs=8 and BPs=4; WM cluster GPs=9 and BPs=3) and 22 older animals (RM cluster GPs=17 and BPs=5; WM cluster GPs=14 and BPs=8). Error bars represent SEM, doted lines represent confidence intervals and continuous lines are linear fits; *$p<0.05$. (WM – working memory; RM – reference memory).

Table S5| Correlations between the performance in the behavioral flexibility task and the dendritic length of mPFC neurons.

			Old animals	Young animals
			**BF**	**BF**
**Cg/PL neurons**	**Apical dendritic length**	Pearson Correlation	-0.042	0.491
		Sig. (2-tailed)	0.836	0.075
		N	27	14
	**Basal dendritic length**	Pearson Correlation	-0.128	-0.422
		Sig. (2-tailed)	0.524	0.133
		N	27	14
**IL neurons**	**Apical dendritic length**	Pearson Correlation	-0.208	**0.611***
		Sig. (2-tailed)	0.340	**0.046**
		N	22	**11**
	**Basal dendritic length**	Pearson Correlation	-0.091	-0.276
		Sig. (2-tailed)	0.679	0.411
		N	22	11

*p<0.05

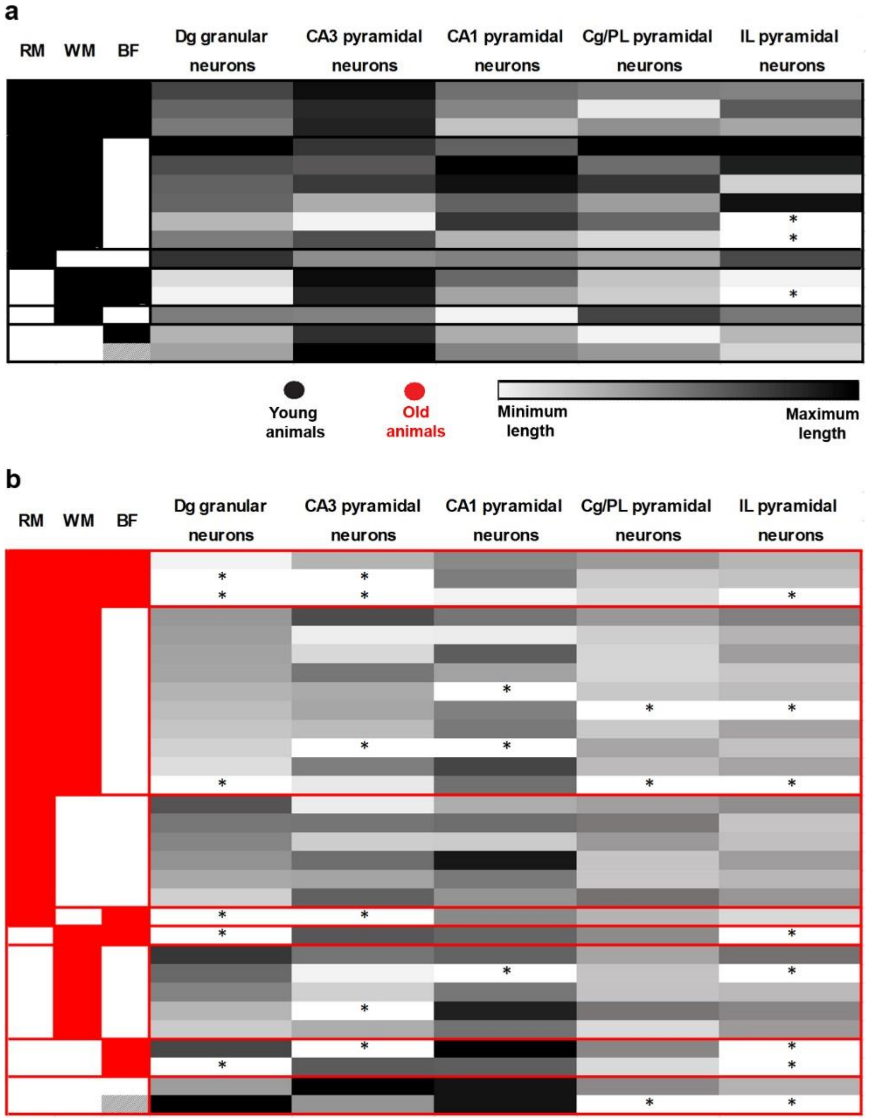

Figure S6| Morphological alterations in the HPC and mPFC of individual young and old animals. A and B) Representation of all the morphological alterations simultaneously occurring in the HPC (Dg granular, CA3 and CA1 pyramidal neurons) and mPFC (Cg/PL and IL pyramidal neurons), regions of both young **(A)** and old group **(B).** Asterisk represents missing analysis. The color scale is relative to the minimum and maximum length within each individual region.

Table S7| List of correlations between dorsal HPC western blot data and the performance in the reference memory, working memory or behavioral flexibility tasks.

				Old animals			Young animals		
				RM	WM	BF	RM	WM	BF
HIPPOCAMPUS	Autophagy	LC3-II	Pearson Correlation	**0.523***	0.288	0.173	**0.790****	0.042	-0.151
			Sig. (2-tailed)	**0.018**	0.218	0.467	**0.004**	0.904	0.677
			N	**20**	20	20	**11**	11	11
		p62	Pearson Correlation	**-0.489***	-0.345	-0.092	-0.012	0.207	-0.291
			Sig. (2-tailed)	**0.029**	0.136	0.699	0.972	0.541	0.415
			N	**20**	20	20	11	11	11
	Dendritic growth	BDNF	Pearson Correlation	**-0.577****	**-0.447***	0.183	0.382	0.610	-0.289
			Sig. (2-tailed)	**0.008**	**0.048**	0.441	0.247	0.061	0.417
			N	**20**	**20**	20	11	11	11
	Synaptic marker	PSD95	Pearson Correlation	**-0.448***	**-0.513***	0.148	0.184	-0.084	-0.058
			Sig. (2-tailed)	**0.048**	**0.021**	0.533	0.588	0.807	0.874
			N	**20**	**20**	20	11	11	10
		Synaptophysin	Pearson Correlation	-0.453	-0.406	0.290	0.286	-0.005	-0.165
			Sig. (2-tailed)	0.052	0.085	0.228	0.394	0.988	0.649
			N	19	19	19	11	11	11
		SNAP25	Pearson Correlation	**-0.560***	-0.339	-0.111	0.208	0.039	-0.342
			Sig. (2-tailed)	**0.010**	0.143	0.641	0.539	0.909	0.334
			N	**20**	20	20	11	11	11

*p<0.05; **p<0.01

Western blot analysis - mPFC

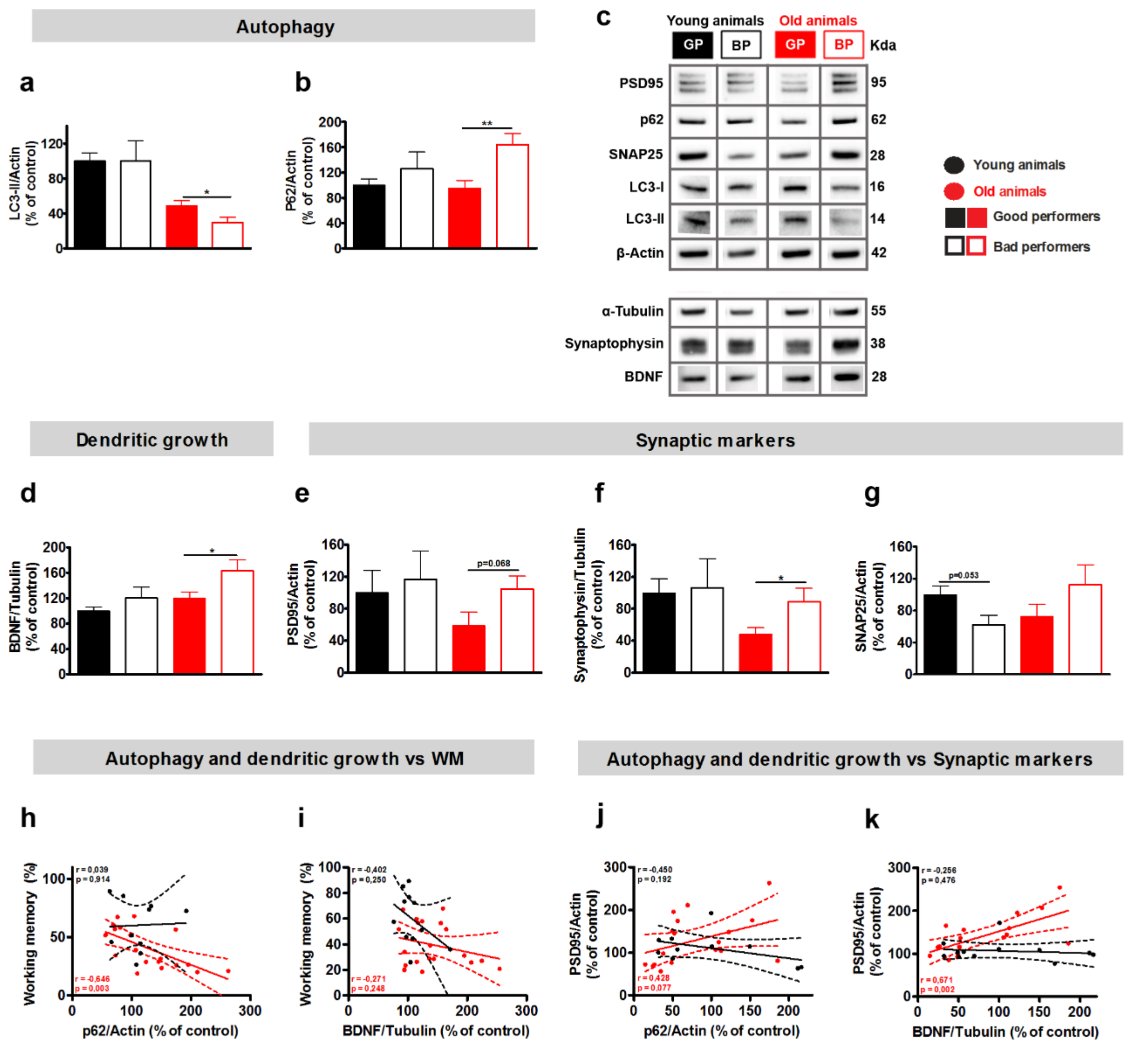

Figure S8| Dysregulation in autophagy signaling and dendritic pruning in the mPFC of older animals. Working memory performance was used to cluster both younger and older animals in GPs and BPs. **A** and **B)** Levels of autophagy markers, LC3-II **(A)** and p62 **(B)**, normalized to actin. **D)** BDNF levels normalized to tubulin. **E, F, G)** Levels of synaptic markers PSD95, SYP and SNAP25 normalized to actin, tubulin, and actin respectively. **C)** Representative western blots of PSD95, p62, SNAP25, LC3, Actin, Tubulin, SYP, and BDNF. For each protein, the blots were cropped from different parts of the same gel. **H** and **I)** Correlation between working memory performance and p62 or BDNF levels, respectively. **J** and **K)** Correlation between PSD95 and p62 or BDNF levels, indicating an association between synaptic marker levels, autophagy and dendritic growth. Number of animals: 10-11

younger animals (GPs=6-7 and BPs=4) and 18-20 older animals (GPs=10 and BPs=8-10). Error bars represent SEM, doted lines represent confidence intervals and continuous lines are linear fits; *p<0.05; **p<0.01; ***p<0.001.

Table S9| Correlations between mPFC western blot data and the performance in the reference memory, working memory or behavioral flexibility tasks.

				Old animals			Young animals		
				**RM**	**WM**	**BF**	**RM**	**WM**	**BF**
**mPFC**	**Autophagy**	**LC3-II**	Pearson Correlation	0.387	0.445	0.003	-0.462	-0.093	0.016
			Sig. (2-tailed)	0.112	0.064	0.989	0.179	0.798	0.968
			N	18	18	18	10	10	10
		**p62**	Pearson Correlation	**-0.504***	**-0.646****	-0.003	-0.573	0.039	-0.444
			Sig. (2-tailed)	**0.028**	**0.003**	0.991	0.083	0.914	0.231
			N	**19**	**19**	19	10	10	10
	**Dendritic growth**	**BDNF**	Pearson Correlation	-0.150	-0.271	-0.203	-0.218	-0.425	-0.099
			Sig. (2-tailed)	0.527	0.248	0.390	0.520	0.193	0.786
			N	20	20	20	11	11	11
	**Synaptic marker**	**PSD95**	Pearson Correlation	-0.113	-0.217	0.010	0.158	-0.020	**0.702***
			Sig. (2-tailed)	0.654	0.386	0.968	0.643	0.954	**0.024**
			N	18	18	18	11	11	**10**
		**Synaptophysin**	Pearson Correlation	-0.199	-0.304	-0.330	-0.262	0.297	0.055
			Sig. (2-tailed)	0.401	0.192	0.155	0.436	0.376	0.879
			N	20	20	20	11	11	11
		**SNAP25**	Pearson Correlation	-0.191	-0.131	0.096	**0.755****	0.449	0.142
			Sig. (2-tailed)	0.434	0.593	0.697	**0.007**	0.166	0.696
			N	19	19	19	**11**	11	11

*p<0.05; **p<0.01

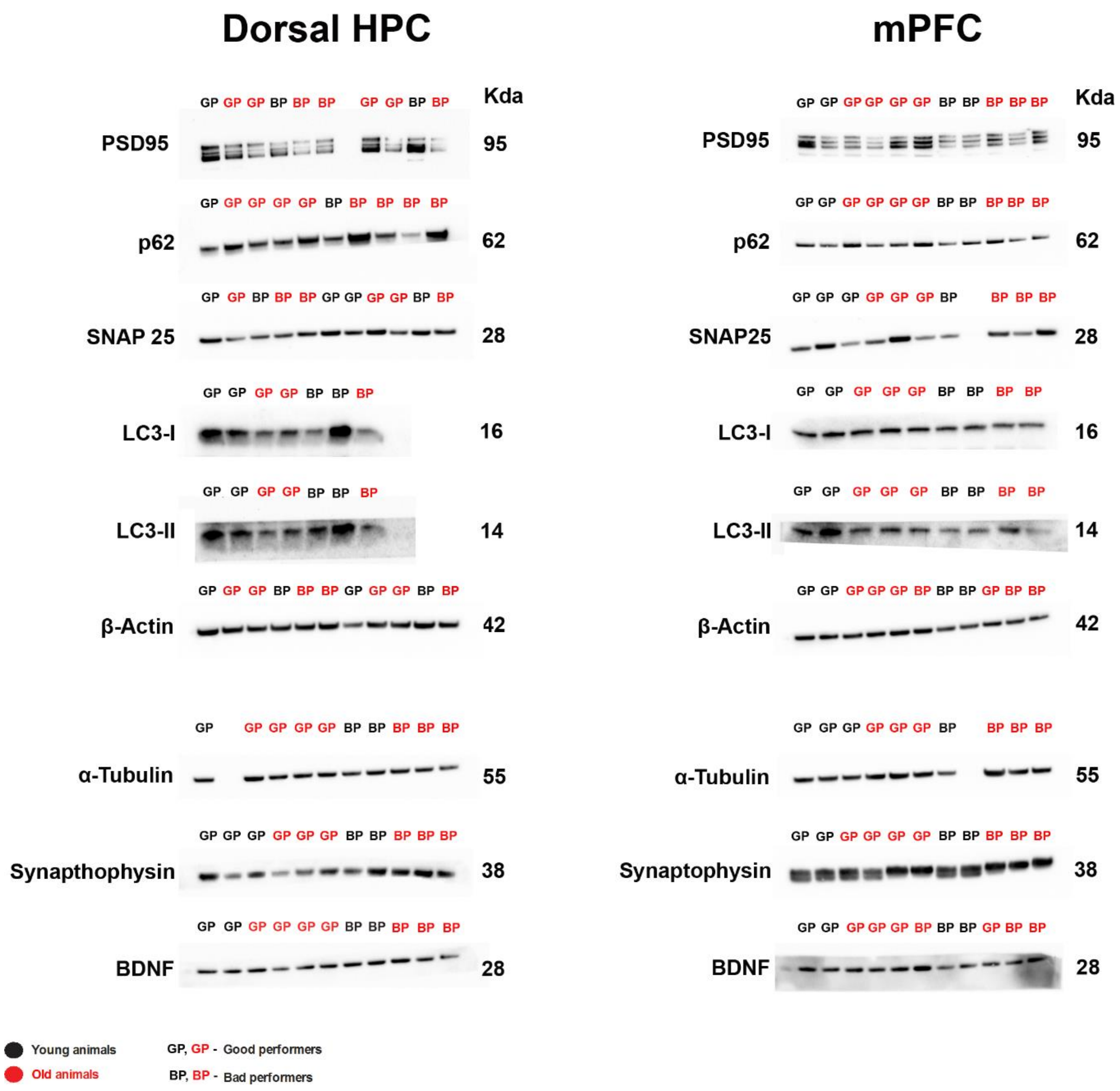

Figure S10| Full length western blots. Full-length western blots for figure 3c and supplementary figure S8c.
